# The Effect of HER2 Status on Gastric Cancer Survival and the Clinical Implications of the HER2-Low Definition: A Retrospective Study

**DOI:** 10.3390/medicina61091675

**Published:** 2025-09-15

**Authors:** Mehmet Uzun, Savas Gokcek, Melis Kilinc, Ferhat Ekinci, Tugay Avci, Atike Pinar Erdogan, Elif Atag, Ilkay Tugba Unek

**Affiliations:** 1 Department of Medical Oncology, Health Sciences University, Tepecik Training and Research Hospital, 35180 Izmir, Turkey; 2 Department of Medical Oncology, Necip Fazıl City Hospital, 46050 Kahramanmaras, Turkey; zorro3563@gmail.com; 3 Department of Internal Medicine, Buca Seyfi Demirsoy Training and Research Hospital, 35360 Izmir, Turkey; drmeliskilinc@gmail.com; 4 Faculty of Medicine, Medical Oncology Department, Manisa Celal Bayar University, 45020 Manisa, Turkey; drferhatekinci@hotmail.com (F.E.); tugayavcii@gmail.com (T.A.); dr_pinarcan@yahoo.com (A.P.E.); 5 Department of Medical Oncology, Dokuz Eylül University, 35340 Izmir, Turkey; elifatag@gmail.com (E.A.); ilkaytugbaunek@gmail.com (I.T.U.)

**Keywords:** advanced gastric cancer, HER-2 expression, HER-2 low, metastatic patterns, overall survival

## Abstract

*Background and Objectives*: HER-2 expression plays a critical role in the biological behavior and treatment of gastric cancer. With the emergence of HER-2-targeted therapies, classification into negative, low, and positive groups has gained clinical importance. The present study focused on assessing the link between HER-2 status and clinical–pathological variables, metastatic involvement, and overall survival (OS) among advanced gastric cancer patients. *Materials and Methods*: A total of 300 patients with advanced gastric adenocarcinoma were retrospectively analyzed. The mean age of the 300 patients included in the study was 61.8 years, and 70% of them were male. Based on immunohistochemistry (IHC) and fluorescence in situ hybridization (FISH), patients were classified as HER-2-negative (IHC 0), HER-2-low (IHC 1+ or 2+/FISH-negative), or HER-2-positive (IHC 3+ or 2+/FISH-positive). Clinicopathological variables, metastatic sites, and OS were compared among groups using Pearson’s Chi-square, Fisher’s exact test, ANOVA, and Kaplan–Meier survival analysis. *Results*: Significant differences were observed among HER-2 subgroups in pathological subtype (*p* = 0.006), liver metastasis (*p* = 0.009), lung metastasis (*p* = 0.006), and other metastatic sites (*p* = 0.001). HER-2-positive patients demonstrated higher rates of adenocarcinoma histology and increased liver and lung metastases. In female patients, HER-2 status was significantly associated with lung (*p* = 0.001) and other metastases (*p* < 0.001). Median OS for the entire cohort was 9.83 months (95% CI: 8.29–11.36). HER-2-positive patients had a significantly longer OS (15.06 months) compared with HER-2-negative patients (8.73 months; *p* = 0.039). *Conclusions*: HER-2 status is an important predictor of metastatic behavior and survival in advanced gastric cancer. HER-2-positive patients display distinct metastatic patterns and improved outcomes, supporting the value of HER-2-targeted therapies. The HER-2-low group may represent a biologically and clinically relevant intermediate subtype requiring further investigation.

## 1. Introduction

Gastric cancer is recognized as the fourth most common cause of cancer-associated death globally and continues to pose a major public health burden, especially in low- and middle-income nations [1]. A large proportion of patients are diagnosed at an advanced stage, limiting therapeutic choices and leading to poorer survival outcomes [2]. Despite recent improvements in survival rates among gastric cancer patients, the prognosis remains unsatisfactory due to the high risk of metastasis. Gastric cancer is a highly heterogeneous disease from histopathological, molecular, and clinical perspectives, and this heterogeneity plays a critical role in both disease progression and treatment response [3].

The expanding application of targeted therapeutic approaches in oncology has underscored the critical role of molecular biomarkers in gastric cancer. Among these, human epidermal growth factor receptor-2 (HER-2) is a membrane-bound tyrosine kinase receptor that regulates cellular growth and differentiation [4]. HER-2 positivity is observed in approximately 10–20% of gastric adenocarcinomas, being more common in intestinal-type tumors, in male patients, and in proximally located tumors [4]. Beyond gastric cancer, HER-2 alterations have been identified across a wide range of solid tumors, with varying prevalence and therapeutic implications. As summarized in Table 1, HER-2 serves as a critical predictive and prognostic biomarker in breast and gastric cancer, while emerging evidence supports its role in colorectal, endometrial, biliary tract, salivary gland, lung, and urothelial cancers. The approval of trastuzumab deruxtecan(T-DXd) in a tumor-agnostic fashion for HER-2-positive (IHC 3+) tumors has further expanded the clinical scope of HER-2-targeted therapy, providing new opportunities for patients with otherwise limited treatment options.

The clinical significance of HER-2 in gastric cancer was established by the ToGA trial (2010), which showed that incorporating trastuzumab into standard chemotherapy regimens significantly improved survival in advanced cases [5]. Based on these findings, HER-2 testing has become one of the standard molecular evaluation methods in advanced gastric cancer. HER-2 assessment is performed not only as positive or negative but also in detail through immunohistochemical (IHC) scoring (0, 1+, 2+, and 3+), leading to the emergence of the concept of “HER-2-low” to describe patients with IHC scores of 1+ or 2+ but FISH-negative [6].

The HER-2-low subtype was first described in breast cancer, where this group—characterized by limited sensitivity to conventional HER-2-targeted therapies—was shown to be responsive to novel antibody–drug conjugates, such as trastuzumab deruxtecan [7]. This finding has suggested that the HER-2-low subgroup in gastric cancer may hold clinical importance, as it could exhibit overlapping molecular features [7,8]. Nevertheless, it is still uncertain if these patients constitute a biologically separate subtype, and the implications of HER-2-low status for metastatic patterns and survival outcomes have not yet been fully elucidated [8].

While some studies have reported that HER-2-low patients demonstrate survival outcomes comparable to those with HER-2 negativity, other investigations have defined this group as a distinct subtype exhibiting clinical features more closely aligned with HER-2 positivity [9]. These conflicting results highlight the necessity for a more comprehensive evaluation of the clinical and prognostic characteristics of HER-2-low gastric cancer patients.

This study aimed to perform a comparative evaluation of clinicopathological characteristics, metastatic distribution, and survival outcomes in advanced gastric cancer patients, stratified by HER-2 status (negative, low, and positive). In this way, the clinical behavior of the HER-2-low subtype can be better understood, and its role within molecular classification and its therapeutic potential can be further assessed.

## 2. Materials and Methods

### 2.1. Patients’ Characteristics and Data Collection

This retrospective study included data from a total of 300 patients who were diagnosed with advanced gastric adenocarcinoma and followed between January 2010 and December 2023 at the Medical Oncology Clinic of Dokuz Eylul University and the Medical Oncology Clinic of Manisa Celal Bayar University Hafsa Sultan Hospital. The diagnosis in all included patients was established through endoscopic biopsy and pathological evaluation, while HER-2 status was determined by IHC and, when necessary, fluorescence in situ hybridization (FISH). HER-2 classification was performed based on IHC results and categorized into three groups: Negative: patients with an IHC score of 0; HER-2-low: patients with an IHC score of 1+ or 2+ and a negative FISH test; HER-2-positive: patients with an IHC score of 3+, or IHC 2+ with a positive FISH test. Inclusion criteria: histopathologically confirmed diagnosis of advanced gastric adenocarcinoma, evaluation of HER-2 status by IHC and/or FISH, and availability of complete clinical follow-up data. Exclusion criteria: presence of a concurrent malignancy, incomplete medical records, or lack of HER-2 assessment. Clinical and demographic data, including age, sex, pathological subtype, metastatic sites, de novo versus secondary metastatic status, and survival outcomes, were retrospectively obtained from patient medical records.

### 2.2. Statistical Analysis

All statistical analyses were conducted with IBM SPSS Statistics for Windows, Version 25.0 (IBM Corp., Armonk, NY, USA). Categorical variables were summarized as numbers and percentages, whereas continuous variables were expressed as mean ± standard deviation (SD) and median (range). For comparisons involving more than two groups, analysis of variance (ANOVA) was applied. The predictive performance of different indices for mortality was assessed using Receiver Operating Characteristic (ROC) curve analysis. Categorical data were compared with the Pearson Chi-square or Fisher’s exact test, as appropriate. Survival differences between groups were examined by the Kaplan–Meier method. A *p*-value < 0.05 was accepted as statistically significant.

## 3. Results

In this study, a total of 300 patients diagnosed with advanced gastric adenocarcinoma between January 2010 and December 2023 and followed at the Medical Oncology Clinic of Dokuz Eylul University and the Medical Oncology Clinic of Manisa Celal Bayar University Hafsa Sultan Hospital were retrospectively reviewed. The average age of the study population was 61.8 ± 11.3 years. Regarding sex distribution, 70% were male (*n* = 210) and 30% were female (*n* = 90).

In subgroup analyses by sex, HER-2-low status in female patients showed a significant correlation with lung metastasis (*p* = 0.001) and with other metastatic sites (*p* < 0.001). Conversely, in male patients, metastatic distribution did not differ significantly according to HER-2 status (*p* > 0.05) (Table 2).

Based on HER-2 IHC scoring and, when necessary, FISH analysis, patients were divided into three groups: HER-2-negative (IHC 0): 211 patients (70.3%); HER-2-low (IHC 1+ or 2+ with FISH-negative): 41 patients (13.7%); HER-2-positive (IHC 3+ or IHC 2+ with FISH-positive): 48 patients (16%). A statistically significant difference was identified between the HER-2 classification groups and the variables of pathological subtype (*p* = 0.006), liver metastasis (*p* = 0.009), lung metastasis (*p* = 0.006), and other metastases (*p* = 0.001).

As shown in Table 3, a statistically significant difference was observed between HER-2 positivity/negativity and the variables of sex (*p* = 0.017), pathological subtype (*p* = 0.010), liver metastasis (*p* = 0.007), lung metastasis (*p* = 0.004), and other metastases (*p* = 0.004).

As shown in Table 4, statistically significant differences were observed between HER-2 score groups and the variables of pathological subtype (*p* = 0.014), liver metastasis (*p* = 0.005), lung metastasis (*p* = 0.014), peritoneal metastasis (*p* = 0.039), and other metastases (*p* = 0.025).

Based on Kaplan–Meier survival analysis, the median OS for the entire cohort was 9.83 months (95% CI: 8.29–11.36). The median OS was 8.89 months for the HER-2-negative group, 11.30 months for the HER-2-low group, and 15.06 months for the HER-2-positive group (Table 5).

A statistically significant survival difference was observed across the groups (*p* = 0.039), with patients harboring HER-2 positivity achieving notably longer survival. While the HER-2-low subgroup had improved survival relative to the HER-2-negative cohort, this finding did not reach conventional statistical significance (*p* = 0.095).

The Cox regression analysis evaluated the impact of clinical and pathological variables on OS. Age (≥65 years) and gender were not significantly associated with survival. Similarly, ECOG performance status ≥ 2 showed a tendency toward worse survival, but the association did not reach statistical significance. Baseline serum albumin ≤ 3.5 g/dL was strongly associated with poorer survival, while higher albumin levels (>3.5 g/dL) were predictive of a significantly reduced risk of death. HER-2 positivity was associated with significantly improved survival compared with HER-2 negativity and this result remained significant in the multivariate model (HR = 0.61, 95% CI = 0.39–0.95; *p* = 0.03). Moreover, patients with de novo metastatic disease had significantly shorter OS compared with those with recurrence. Recurrence remained an independent predictor of favorable survival in the multivariate analysis (HR = 0.46, 95% CI = 0.35–0.60; *p* < 0.001) (Table 6).

## 4. Discussion

Establishing HER-2 expression is vital in the therapeutic management of gastric carcinoma; however, its influence on prognosis remains inconclusive. In a systematic review, while 2studies reported significantly longer survival in patients with HER-2 overexpression, 13 studies presented the opposite findings [10]. Acknowledging the contradictory findings concerning the prognostic impact of HER-2, our investigation focused on evaluating the influence of different HER-2 categories (negative, low, and positive) on clinical and pathological characteristics, metastatic spread, and overall survival in patients with advanced gastric adenocarcinoma.

Our findings demonstrated that HER-2-positive patients had higher rates of liver and lung metastases, as well as significantly longer overall survival. Although the HER-2-low subgroup showed improved survival relative to the HER-2-negative cohort, the finding was limited to borderline statistical significance. These results suggest that HER-2 status represents an important biomarker influencing both metastatic behavior and survival in advanced gastric cancer [11,12].

In our study, the frequency of HER-2-low was 13.7%, whereas Kim et al. reported this rate as 41%, attributing the higher prevalence to the larger sample size and the evaluation of HER-2 status in all surgical specimens [13]. A smaller-scale study documented a 28% frequency of HER-2-low expression, identified through postoperative specimen evaluation [14]. Although the prognosis of metastatic gastric cancer remains poor, chemotherapy significantly improves survival, with median overall survival generally expressed in months regardless of the chemotherapy regimen used. The ToGA study helped define the clinical significance of HER-2-positive gastric carcinoma. Its results showed that incorporating trastuzumab into standard chemotherapy conferred a modest extension in survival, with a mean increase of 2.7 months; however, a posthoc analysis demonstrated a greater improvement of 4.2 months in median overall survival [5]. Although the prognostic effect of HER-2 status was not formally assessed in the ToGA study, interestingly, the median survival times in the control arm appeared to increase in correlation with HER-2 protein expression levels. The prognostic role of HER-2 expression in metastatic cases untreated with HER-2-targeted agents remains unclear.

Our results indicated a significantly prolonged median OS for the HER-2-positive subgroup (15.06 months) relative to the HER-2-negative cohort (8.73 months). Comparable outcomes have been described in earlier investigations [15,16]. Likewise, in a large retrospective study, patients with HER-2-positive gastric cancer were shown to have significantly longer survival and to develop liver metastases more frequently [17].

When evaluated in terms of metastatic patterns, our study demonstrated that HER-2-positive patients had higher rates of liver (*p* = 0.009) and lung (*p* = 0.006) metastases. These findings are consistent with previous evidence suggesting that HER-2 may enhance the invasive properties of tumor cells and increase their potential for distant organ dissemination [18]. In particular, hepatotropism in HER-2-positive gastric cancers has been frequently reported in previous autopsy series and imaging studies [19].

In recent years, the concept of HER-2-low has begun to emerge in the gastric cancer literature following its introduction in breast cancer. While HER-2-directed treatments are established for metastatic gastric cancer with HER-2 positivity, the HER-2-low cohort remains without specific therapeutic strategies. Novel therapeutic targets are currently among the most widely investigated areas. The HER-2-low group represents a subset traditionally considered HER-2-negative but characterized by low-level HER-2 expression, and it has been shown to potentially respond to next-generation antibody–drug conjugates, such as trastuzumab deruxtecan [7]. In subgroup analyses of the DESTINY-Gastric01 trial, patients with HER-2-low gastric cancer were also reported to derive benefit from trastuzumab deruxtecan treatment [20].

Our results revealed a survival advantage in the HER-2-low group compared with HER-2-negative patients, though the difference did not reach statistical significance (*p* = 0.095). Such a trend may indicate that HER-2-low tumors occupy an intermediate position with respect to clinical behavior [21]. Conversely, in the study by Unal et al., no significant difference in OS was demonstrated between HER-2-low and HER-2-negative tumors, with survival times ranging between 10 and 12 months [22]. The biological behavior of HER-2-low gastric cancer patients is heterogeneous. Some subgroups resemble HER-2-negative tumors, whereas others may exhibit a survival pattern more similar to HER-2-positive tumors [23]. In our study, lung and other metastasis rates were found to be higher in HER-2-low patients, particularly among females. Such sex-based differences have been investigated in only a limited number of studies, and it has been suggested that hormonal, molecular, or immunological variations may account for these findings [24]. In females, the role of hormonal factors in modulating HER-2 expression and shaping the tumor microenvironment warrants further investigation in gastric cancer, a concept largely inspired by insights gained from the breast cancer literature.

In addition to HER-2, microsatellite instability (MSI) represents an important biomarker in gastric cancer. Its evaluation on biopsy samples during the diagnostic phase enables timely therapeutic strategies, especially in the context of immunotherapy. This highlights the complementary role of MSI and HER-2 testing in guiding precision oncology approaches [25].In recent years, immunotherapy, particularly in combination with anti-HER-2 therapies in HER-2-positive gastric cancer, has been the focus of increasing research. The KEYNOTE-811 trial has provided promising data indicating a synergistic effect between checkpoint inhibitors and trastuzumab-based treatments. In this context, the role of immunotherapy in HER-2-positive patients may become increasingly significant in future standard treatment approaches [26]. In this context, the role of immunotherapy in HER-2-positive patients may gain greater importance in future standard treatment approaches.

Our study has certain limitations. The retrospective design, the relatively small sample size in some subgroups, and the heterogeneity of treatment regimens may limit the generalizability of the results. In addition, the heterogeneity of the HER-2-low group and inter-test variability in interpretation should also be taken into consideration.

In future prospective studies, the treatment response, molecular profile differences, and immune microenvironmental associations of the HER-2-low group should be investigated in greater detail. In particular, randomized controlled trials evaluating agents such as trastuzumab deruxtecan in HER-2-low gastric cancer will provide valuable guidance. Therefore, it has been proposed that the HER-2-low subtype should be regarded not merely as a level of expression but also as a relevant factor in therapeutic targeting and biological subtype classification.

## 5. Conclusions

Our findings indicate that HER-2 status may serve not only as a determinant for targeted therapy decisions but also as an important marker for predicting metastatic behavior and prognosis. This underscores the importance of considering the HER-2-low group as a distinct biological subtype, particularly in clinical research.

## Figures and Tables

**Table 1:** Role of HER-2 across different cancer types and therapeutic implications.

Cancer Type	Prevalence of HER-2 Alterations	Clinical/Biological Role of HER-2	Therapeutic Approaches
Breast Cancer	15–20% HER-2-positive; ~45–55% HER-2-low	Driver of aggressive biology; strong predictive marker	Trastuzumab, Pertuzumab, T-DM1, Trastuzumab deruxtecan(T-DXd), Tucatinib
Gastric/GEJ Adenocarcinoma	10–20% HER-2-positive; ~15% HER-2-low	Prognostic/predictive biomarker; defines therapeutic subgroup	Trastuzumab (1L); Pembrolizumab + Trastuzumab + Chemo (1L); T-DXd(2L+)
Colorectal Cancer (mCRC, RAS WT)	~3–5% HER-2-positive	Predicts resistance to anti-EGFR; targetable in WT	Tucatinib + Trastuzumab; T-DXd(investigational)
Endometrial Serous Carcinoma	25–30% HER-2-positive	Driver in aggressive serous subtype	Trastuzumab + Carbo/Taxane
NSCLC (HER-2mutant)	2–4% HER-2 mutations; <2% amplification	Oncogenic driver	T-DXd; HER-2-TKIs (investigational)
Biliary Tract Cancer	5–15% HER-2 alterations	Emerging biomarker; associated with poor prognosis	Zanidatamab; T-DXd
Salivary Duct Carcinoma	Amplification 12–52%; overexpression 17–44%	Strong biomarker in aggressive histology	Trastuzumab, Pertuzumab, T-DM1, T-DXd
Ovarian Cancer	7–12% (esp. serous subtypes)	Potential driver in subsets	Trastuzumab; ADCs (trials)
Cervical Cancer	~5–10% overexpression (rare)	Potential prognostic/predictive role	T-DXd(pan-tumor option)
Urothelial (Bladder) Cancer	~5% HER-2-positive	Exploratory role; resistance pathways	T-DXd; Disitamabvedotin(China)
Prostate Cancer	Variable; up to ~10% overexpression/signaling activation	Linked to castration-resistant disease biology	Investigational HER-2 inhibitors/ADCs
Pancreatic Cancer	2–5% amplification	Rare driver in aggressive subgroup	T-DXd(trial evidence)

ADC = Antibody–Drug Conjugate; Carbo = Carboplatin; Chemo = Chemotherapy; GEJ = Gastroesophageal Junction; HER-2 = Human Epidermal Growth Factor Receptor-2; NSCLC = Non-Small-Cell Lung Cancer; T-DM1 = Trastuzumab Emtansine; T-DXd = Trastuzumab Deruxtecan; TKI = Tyrosine Kinase Inhibitor; WT = Wild-Type; 1L = First Line.

**Table 2:** Comparison of sociodemographic and clinical characteristics according to HER-2 classification groups.

	HER-2 Classification			
Variables	HER-2-Negative (HER-2 0) *n* = 211	HER-2 (Low)1 or 2-Positive FISH-Negative *n* = 41	HER-2 3-Positive or HER-2 2+ FISH-Positive *n* = 48	*p*
Age				
≤65	130 (61.6)	28 (68.3)	34 (70.8)	0.402 ^a^
>65	81 (38.4)	13 (31.7)	14 (29.2)	
Gender				
Man	139 (65.9)	27 (65.9)	40 (83.3)	0.057 ^a^
Woman	72 (34.1)	14 (34.1)	8 (16.7)	
Pathological Subtype				
Adenocancer	144 (68.2)	35 (85.4)	44 (91.7)	**0.006** ^a^
Signet Ring Cell	40 (19)	4 (9.8)	3 (6.3)	
Poorly Cohesive Carcinoma	27 (12.8)	2 (4.9)	1 (2.1)	
Denovo Status				
Recurrence	59 (28)	17 (41.5)	14 (29.2)	0.223 ^a^
De novo Metastatic	152 (72)	24 (58.5)	34 (70.8)	
Liver Metastasis				
No	144 (68.2)	23 (56.1)	22 (45.8)	**0.009** ^a^
Yes	67 (31.8)	18 (43.9)	26 (54.2)	
Lung Metastasis				
No	184 (87.2)	32 (78)	33 (68.8)	**0.006** ^a^
Yes	27 (12.8)	9 (22)	15 (31.3)	
Bone Metastasis				
No	173 (82)	36 (87.8)	38 (79.2)	0.551 ^a^
Yes	38 (18)	5 (12.2)	10 (20.8)	
Distant LN Metastasis				
No	122 (57.8)	20 (48.8)	24 (50)	0.408 ^a^
Yes	89 (42.2)	21 (51.2)	24 (50)	
Peritoneal Metastasis				
No	105 (49.8)	23 (56.1)	27 (56.3)	0.597 ^a^
Yes	106 (50.2)	18 (43.9)	21 (43.8)	
Other Metastasis				
No	149 (70.6)	20 (48.8)	30 (62.5)	**0.001** ^a^
Extracranial	59 (28)	20 (48.8)	13 (27.1)	
Cranial	3 (1.4)	1 (2.4)	5 (10.4)	

HER-2: Human Epidermal Growth Factor Receptor-2; FISH: Fluorescence In Situ Hybridization; LN: Lymph Node; ^a^: Pearson Chi-square Test.

**Table 3:** Comparison of sociodemographic and clinical characteristics according to HER-2 status groups.

	HER-2 Status		
Variables	Negative *N* = 252	Positive *N* = 48	*p*
Age			
≤65	158 (62.7)	34 (70.8)	0.282 ^a^
>65	94 (37.3)	14 (29.2)	
Gender			
Man	166 (65.9)	40 (83.3)	**0.017** ^a^
Woman	86 (34.1)	8 (16.7)	
Pathological Subtype			
Adenocancer	179 (71)	44 (91.7)	**0.010** ^a^
Signet Ring Cell	44 (17.5)	3 (6.3)	
Poorly Cohesive Carcinoma	29 (11.5)	1 (2.1)	
Denovo Status			
Recurrence	76 (30.2)	14 (29.2)	0.891 ^a^
De novo Metastatic	176 (69.8)	34 (70.8)	
Liver Metastasis			
No	167 (66.3)	22 (45.8)	**0.007** ^a^
Yes	85 (33.7)	26 (54.2)	
Lung Metastasis			
No	216 (85.7)	33 (68.8)	**0.004** ^a^
Yes	36 (14.3)	15 (31.3)	
Bone Metastasis			
No	209 (82.9)	38 (79.2)	0.530 ^a^
Yes	43 (17.1)	10 (20.8)	
Distant LN Metastasis			
No	142 (56.3)	24 (50)	0.417 ^a^
Yes	110 (43.7)	24 (50)	
Peritoneal Metastasis			
No	128 (50.8)	27 (56.3)	0.488 ^a^
Yes	124 (49.2)	21 (43.8)	
Other Metastasis			
No	169 (67.1)	30 (62.5)	**0.004** ^a^
Extracranial	79 (31.3)	13 (27.1)	
Cranial	4 (1.6)	5 (10.4)	

HER-2: Human Epidermal Growth Factor Receptor-2; LN: Lymph Node; ^a^: Pearson Chi-square Test.

**Table 4:** Comparison of sociodemographic and clinical characteristics according to HER-2 score groups.

	HER-2 Score				
Variables	0 *N* = 211	1+ *N* = 30	2+ *N* = 21	3+ *N* = 38	*p*
Age					
≤65	130 (61.6)	23 (76.7)	10 (47.6)	29 (76.3)	0.056 ^a^
>65	81 (38.4)	7 (23.3)	11 (52.4)	9 (23.7)	
Gender					
Man	139 (65.9)	21 (70)	14 (66.7)	32 (84.2)	0.165 ^a^
Woman	72 (34.1)	9 (30)	7 (33.3)	6 (15.8)	
Pathological Subtype					
Adenocancer	144 (68.2)	25 (83.3)	20 (95.2)	34 (89.5)	**0.014** ^b^
Signet Ring Cell	40 (19)	4 (13.3)	0 (0)	3 (7.9)	
Poorly Cohesive Carcinoma	27 (12.8)	1 (3.3)	1 (4.8)	1 (2.6)	
Denovo Status					
Recurrence	59 (28)	12 (40)	10 (47.6)	9 (23.7)	0.129 ^a^
De novo Metastatic	152 (72)	18 (60)	11 (52.4)	29 (76.3)	
Liver Metastasis					
No	144 (68.2)	16 (53.3)	14 (66.7)	15 (39.5)	**0.005** ^a^
Yes	67 (31.8)	14 (46.7)	7 (33.3)	23 (60.5)	
Lung Metastasis					
No	184 (87.2)	24 (80)	14 (66.7)	27 (71.1)	**0.014** ^a^
Yes	27 (12.8)	6 (20)	7 (33.3)	11 (28.9)	
Bone Metastasis					
No	173 (82)	26 (86.7)	19 (90.5)	29 (76.3)	0.511 ^a^
Yes	38 (18)	4 (13.3)	2 (9.5)	9 (23.7)	
Distant LN Metastasis					
No	122 (57.8)	14 (46.7)	12 (57.1)	18 (47.4)	0.486 ^a^
Yes	89 (42.2)	16 (53.3)	9 (42.9)	20 (52.6)	
Peritoneal Metastasis					
No	105 (49.8)	13 (43.3)	17 (81)	20 (52.6)	**0.039** ^a^
Yes	106 (50.2)	17 (56.7)	4 (19)	18 (47.4)	
Other Metastasis					
No	149 (70.6)	15 (50)	13 (61.9)	22 (57.9)	**0.025** ^b^
Extracranial	59 (28)	14 (46.7)	6 (28.6)	13 (34.2)	
Cranial	3 (1.4)	1 (3.3)	2 (9.5)	3 (7.9)	

HER-2: Human Epidermal Growth Factor Receptor-2; LN: Lymph Node; ^a^: Pearson Chi-square Test; ^b^: Fisher’s Exact Test. *p* < 0.05 was considered statistically significant.

**Table 5:** Comparison of overall survival among patients.

Variables	2-Year Survival%	5-Year Survival%	Median (95% CI)	*p*
All cohort	12.6	3.4	9.83 (8.29–11.36)	
HER-2-Low Classification				
HER-2-Negative (HER-2 0)	10.5	3.5	8.73 (7.34–10.12)	0.095
HER-2-Low (IHC 1+ or 2+, FISH-negative)	13.9	2.8	11.30 (5.57–17.03)	
HER-2-Positive (IHC 3+ or IHC 2+ with FISH-positive)	20.4	3.4	15.06 (11.02–19.10)	
HER-2Status				
Negative	10.6	3.4	9.03 (7.62–10.44)	**0.039**
Positive	20.4	3.4	15.06 (11.02–19.10)	
HER-2Score				
0	10.5	3.5	8.73 (7.34–10.12)	0.271
1+	12.0	4.0	13.13 (9.51–16.75)	
2+	20.5	-	12.56 (5.86–19.26)	
3+	19.8	5.0	14.30 (9.03–19.56)	

HER-2: Human Epidermal Growth Factor Receptor-2; Kaplan–Meier Curve; Log-rank Test. *p* < 0.05 was considered statistically significant.

**Table 6:** Univariate and multivariate Cox regression analysis for overall survival.

Characteristics	*n* (%)	Median Overall Survival	Univariate Analyses	*p*-Value	Multivariate Analyses	*p*-Value
			HR (95% CI)		HR (95% CI)	
Age						
<65	182 (60.6)	13	1			
≥65	118 (39.4)	14	1.11 (0.87–1.41)	0.38		
Gender						
Man	206 (68.6)	14	1			
Woman	94 (31.4)	14	1.04 (0.80–1.34)	0.75		
Baseline Albumin at Diagnosis						
≤3.5	96 (32)	10	1			
>3.5	204 (68)	16	0.64 (0.49–0.82)	**<0.001**		
ECOG Status						
Ecog 0/1	255 (85)	14	1			
Ecog ≥ 2	45 (15)	12	1.04 (0.75–1.45)	0.78		
Pathological Subgroup						
Adenocancer	223 (74.3)	15	1			
The Others	77 (25.7)	12	1.27 (0.97–1.67)	0.71		
HER-2 Status						
Negative	252 (84)	13	1			
Positive	48 (16)	22	0.65 (0.47–0.90)	**0.01**	0.61 (0.39–0.95)	**0.03**
According to HER-2-Low Status						
HER-2-Negative	211 (70.3)	13	1			
HER-2 (low)	41 (13.6)	15	1			
HER-2 3+	48 (16)	22	1.56 (1.12–2.18)	**0.008**		
Denovo Status						
Denovo Metastatic	210 (70)	11	1		1	
Recurrence	90 (30)	23	0.47 (0.36–0.61)	**<0.001**	0.46 (0.35–0.60)	**<0.001**

ECOG: Eastern Cooperative Oncology Group; HER-2: Human Epidermal Growth Factor Receptor 2.

## Data Availability

Due to patient rights and confidentiality regulations in our country, data sharing cannot be conducted directly, but upon request, data can be sent after consultation with the authors and the ethics committee.
